# First person – Myriam de Graaf and Juul Hubert

**DOI:** 10.1242/bio.045138

**Published:** 2019-06-15

## Abstract

First Person is a series of interviews with the first authors of a selection of papers published in Biology Open, helping early-career researchers promote themselves alongside their papers. Myriam de Graaf and Juul Hubert are co-first authors on ‘Influence of arm swing on cost of transport during walking’, published in BiO. Myriam and Juul are both (research) master's students in the lab of Andreas Daffertshofer and Raôul Oudejans–John van der Kamp at the Faculty of Behavioural and Human Movement Sciences, Vrije Universiteit, Amsterdam, investigating human movement sciences. Myriam's interest lies predominantly in motor control, neuroscience and biomechanics, while Juul is focused on applications in (elite) sport.

**What is your scientific background and the general focus of your lab?**

We have both completed a bachelor's degree in human movement sciences at the Vrije University (VU) Amsterdam. This is a study program that takes a broad look at all aspects concerning human movement, from biomechanics to psychology and from neuroscience to physiology. Our bachelor's thesis was on the influence of arm swing on cost of transport during walking. Last year we improved our research methodology in a few areas and executed the study again, leading to this publication. At the moment we are both master's students in human movement sciences at VU Amsterdam.


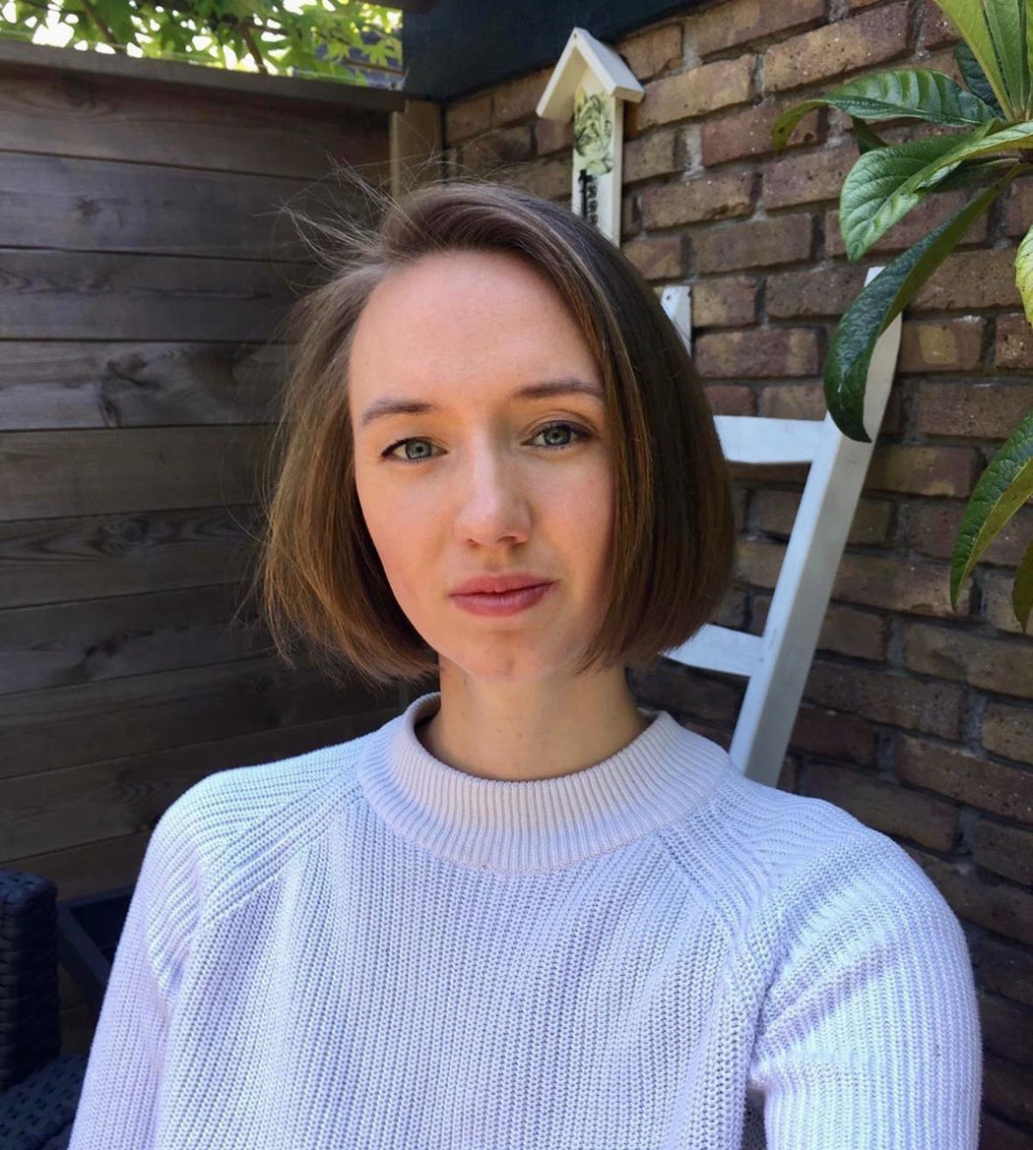


**Myriam de Graaf**

MdG: During my bachelor's, I focused on motor control and muscle synergies for two other major projects. For my master's thesis I've shifted toward a more neuroscientific topic, as I am attempting to predict and record signal propagation in the spinal cord.


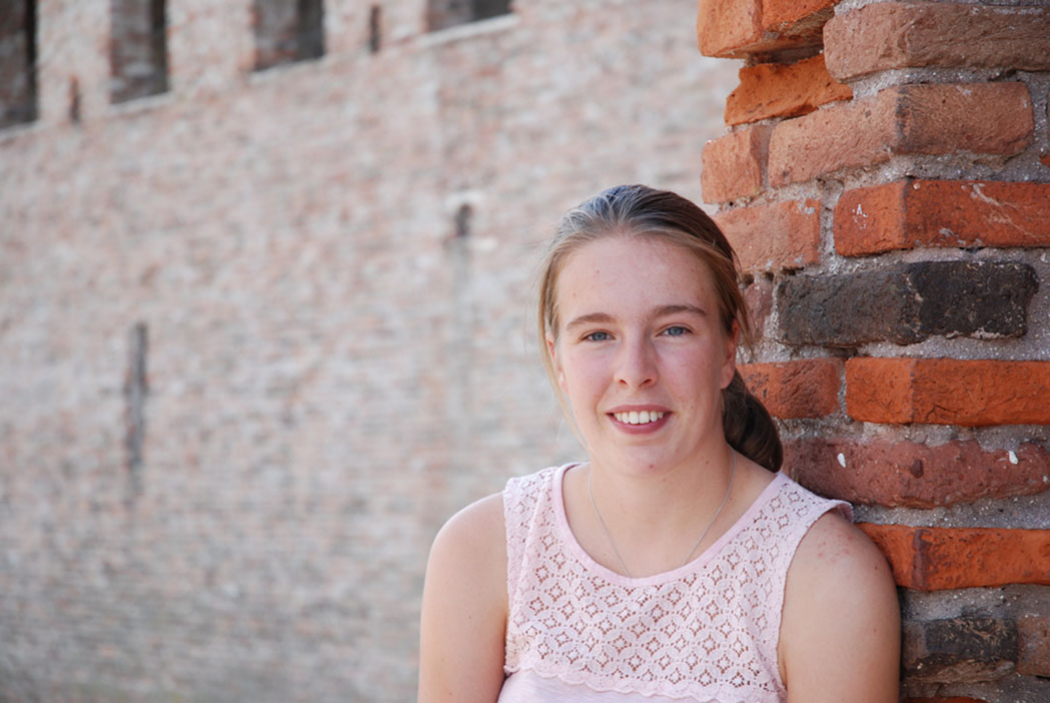


**Juul Hubert**

JH: The literature review I wrote during my bachelor's was about the difference in physical performance of elite athletes between hypobaric hypoxia and normobaric hypoxia. This year during my master's degree, I'm examining the cognitive performances of elite athletes in extreme conditions: high temperature and high relative humidity.

**How would you explain the main findings of your paper to non-scientific family and friends?**

We investigated the influence of arm swing amplitude on the amount of energy that is consumed during walking. Previous studies have already shown that swinging our arms reduces energy consumption compared to walking without swinging our arms. This is most likely because arm swing counteracts the movement of the legs, thereby decreasing the amount our body rotates (i.e. it decreases the vertical angular momentum). This means our legs need to perform less work to counteract the rotation when we want to switch stance feet (i.e. there is a decreased ground reaction moment). However, it seemed that the rotation caused by the legs was not counteracted completely. Therefore, we thought, what if we increased the amplitude of our arm swing? Could that lead to an increased compensation of the rotation caused by the legs? And could this in turn decrease energy consumption even further? Our results show that increasing the arm swing amplitude does decreases the rotation (vertical angular momentum) of our body, but not the energy consumption. Our study indicates that we walk with (near) optimal arm swing amplitude already.

**What are the potential implications of these results for your field of research?**

Based on the current results, changing our arm swing amplitude does not decrease our energy consumption. However, our results also show that the ground reaction moment does decrease with higher arm swing amplitudes. So, walking with an exaggerated arm swing could be beneficial for those who want to decrease this GRM, for instance to alleviate the legs in lower extremity disorders. As a side note, for anybody who wants to burn some extra calories while walking it does appear to be beneficial to change arm swing amplitude.

“For anybody who wants to burn some extra calories while walking it does appear to be beneficial to change arm swing amplitude.”

**What has surprised you the most while conducting your research?**

While our hypothesis predicted a potential decrease in energy consumption when swinging our arms, within the concept of metabolically-optimized walking, one would expect that any deviation from a self-chosen gait would actually increase the energy costs. Our results, however, did not show an increase in energy consumption with a slightly exaggerated arm swing. In fact, the energy consumption was even slightly lower. While this difference was not significant, and might even be due to measurement artefacts, we were still surprised to see it.

**What, in your opinion, are some of the greatest achievements in your field and how has this influenced your research?**

MdG: Since we are still Msc students, we do not have a very specialized field of study yet. Human movement sciences – the name of our Bsc and Msc studies – is a very broad field of study that covers everything related to human movement, from psychology to biomechanics and from tissue engineering to neuroscience. Therefore, it is hard to pinpoint just one (or a few) great achievements. One of the papers I remember reading during my Bsc that I think had a lot of impact was an early design for a force plate, something that is almost omnipresent in human movement sciences (or at least biomechanics) nowadays. I think the design of such key pieces of equipment are the greatest achievements, since they have enabled the execution of many other important studies. More recently, I think machine learning techniques could fulfill such a role in improving our understanding of the functioning of our bodies (especially in neuroscience). It has not yet made its introduction into the core curriculum with in our Bsc or Msc programs, but I can see that changing not too long from now.

JH: Human movement scientists dare to think outside the box. For example, all speedskaters used skates with fixed metal blades. However, human movement scientists developed the klapskate because their knowledge told them speedskaters could skate faster. This way of thinking is very important in our field to develop new things and improve existing ones to perform better in both sport and rehabilitation.

“I think the design of […] key pieces of equipment are the greatest achievements, since they have enabled the execution of many other important studies.”

**Figure:**
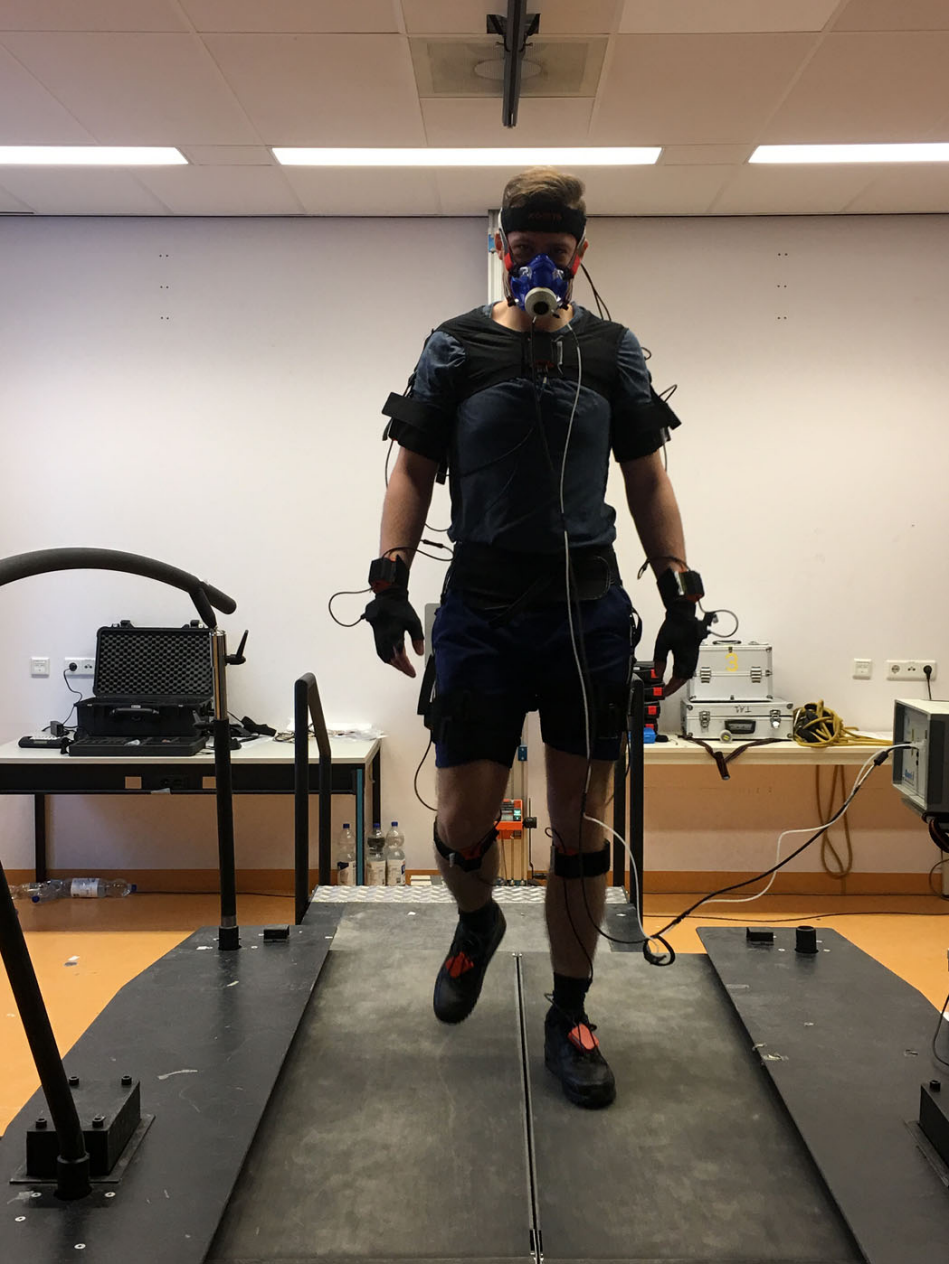
**Subject walking on the Dual Belt while wearing the Xsens suit.**

**What changes do you think could improve the professional lives of early-career scientists?**

MdG: Since I am still a student, I do not yet have a complete image of life as a scientist. For me, the main problem right now is funding. I'd love to continue the research I'm doing during my master's into a PhD project, but it's not easy to find a way to finance this. Most grants I found required several years of research experience to apply. I think this makes early-career scientists very dependent on their PIs. Opening up more grants for starting researchers might not be a bad thing.

**What's next for you?**

MdG: Currently, I'm working on my Msc thesis, where I am trying to record somatosensory evoked potentials over the spinal cord using high-density electromyography. I am aiming to obtain my Msc degree this summer and hope to find a PhD position in a related field.

JH: Like Myriam, right now I am working on my thesis to obtain my Msc degree. It is about the effects of a high ambient temperature in combination with a high relative humidity on the cognitive functioning of elite athletes. My results will be used by the Dutch Olympic Team to optimally prepare for the Olympic Summer Games Tokyo, Japan. I am trying to finish my master's degree by end of this year, 2019. After finishing, I will try to find a job where I can apply my knowledge about human movement sciences in the (elite) sport context.
